# Large Orbital Moment of Two Coupled Spin-Half Co Ions in a Complex on Gold

**DOI:** 10.1021/acsnano.3c01595

**Published:** 2023-05-24

**Authors:** Chao Li, Roberto Robles, Nicolas Lorente, Sanjoy Kr Mahatha, Sebastian Rohlf, Kai Rossnagel, Alessandro Barla, Boris V. Sorokin, Stefano Rusponi, Philippe Ohresser, Sara Realista, Paulo N. Martinho, Torben Jasper-Toennies, Alexander Weismann, Richard Berndt, Manuel Gruber

**Affiliations:** † Institut für Experimentelle und Angewandte Physik, Christian-Albrechts-Universität zu Kiel, 24098 Kiel, Germany; ‡ Centro de Física de Materiales CFM/MPC (CSIC-UPV/EHU), 20018 Donostia-San Sebastián, Spain; § Donostia International Physics Center (DIPC), 20018 Donostia-San Sebastian, Spain; ∥ Ruprecht Haensel Laboratory, Deutsches Elektronen-Synchrotron DESY, 22607 Hamburg, Germany; ⊥ Istituto di Struttura della Materia (ISM), Consiglio Nazionale delle Ricerche (CNR), 34149 Trieste, Italy; # Institute of Physics, Ecole Polytechnique Fédérale de Lausanne (EPFL), Station 3, 1015 Lausanne, Switzerland; ∇ Synchrotron SOLEIL, L’Orme des Merisiers, 91190 Saint Aubin, France; ◆ Centro de Química Estrutural, Institute of Molecular Sciences, Departamento de Química e Bioquímica, Faculdade de Ciências, Universidade de Lisboa, Campo Grande, 1749-016 Lisboa, Portugal; ¶ Faculty of Physics and CENIDE, University of Duisburg-Essen, 47057 Duisburg, Germany

**Keywords:** dinuclear complex, orbital moment, magnetic anisotropy, exchange coupling, scanning tunneling microscopy, X-ray magnetic circular dichroism, density functional theory

## Abstract

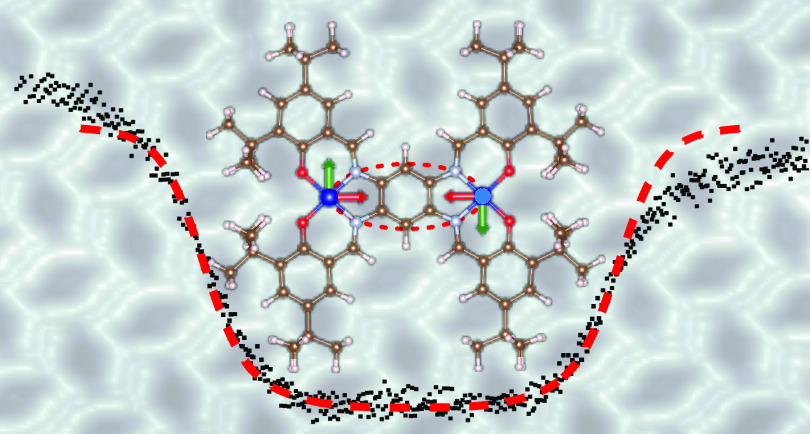

The magnetic properties of transition-metal ions are generally described by the atomic spins of the ions and their exchange coupling. The orbital moment, usually largely quenched due the ligand field, is then seen as a perturbation. In such a scheme, *S* = 1/2 ions are predicted to be isotropic. We investigate a Co(II) complex with two antiferromagnetically coupled 1/2 spins on Au(111) using low-temperature scanning tunneling microscopy, X-ray magnetic circular dichroism, and density functional theory. We find that each of the Co ions has an orbital moment comparable to that of the spin, leading to magnetic anisotropy, with the spins preferentially oriented along the Co–Co axis. The orbital moment and the associated magnetic anisotropy is tuned by varying the electronic coupling of the molecule to the substrate and the microscope tip. These findings show the need to consider the orbital moment even in systems with strong ligand fields. As a consequence, the description of *S* = 1/2 ions becomes strongly modified, which have important consequences for these prototypical systems for quantum operations.

The orbital momentum of transition metal ions strongly depends on the atom coordination and rapidly quenches to the negligible values in bulk.^1^ The magnetic properties of solids and molecules are therefore mainly determined by the atomic spins and their exchange coupling. The effect of the remaining orbital moment may be treated as a perturbation and is at the origin of magnetic anisotropy, which has been extensively studied for atoms and clusters on surfaces.^2−6^ The magnetic properties of atoms are consequently often modeled with a spin Hamiltonian that predicts the absence of magnetic anisotropy for spin-half ions.

Liu et al.^7^ and Whangbo^8^ et al. used density functional theory calculations to analyze the magnetic anisotropy of spin-1/2 Cu^2+^ ions in crystals and suggested that previous interpretations of the anisotropy in terms of anisotropic exchange coupling or magnetic dipole–dipole interactions were not complete. They rather emphasized the importance of spin–orbit coupling. Exchange coupling in crystals can be complex and consequently it is desirable to investigate the magnetic anisotropy of two exchange-coupled spin-1/2 ions in a fairly simple ligand field. Dinuclear (or polynuclear) molecular compounds, which are intensively investigated in the gas-phase,^9−13^ may serve as model systems. Deposition of such complexes on surfaces has so far led to fragmentation^14,15^ or to apparent quenching of the internuclear exchange coupling because of excessive interaction with the substrate.^16−18^ Magnetic excitations have been observed for bulky three-dimensional Mn_12_ and Fe_4_^19,20^ complexes on ultrathin insulator layers.

We report on the planar dicobalt complex C_66_H_86_Co_2_N_4_O_4_ (di-Co, Figure 1a) composed of two spin-1/2 ions adsorbed on Au(111). Combining STM, X-ray magnetic circular dichroism (XMCD), and density functional theory (DFT), we show that each of the two Co ions has an orbital moment of similar magnitude as the spin moment. This orbital moment, through spin–orbit coupling, causes magnetic anisoptropy. The spins within this complex are antiferromagnetically coupled and oriented along the Co–Co axis. We further observed that the magnetic anisotropy of the system may be tuned by varying the electronic coupling of the Co(II) ion with the substrate through manipulation of peripheral *tert*-butyl groups or by reducing the tip-molecule distance.

**Figure 1:**
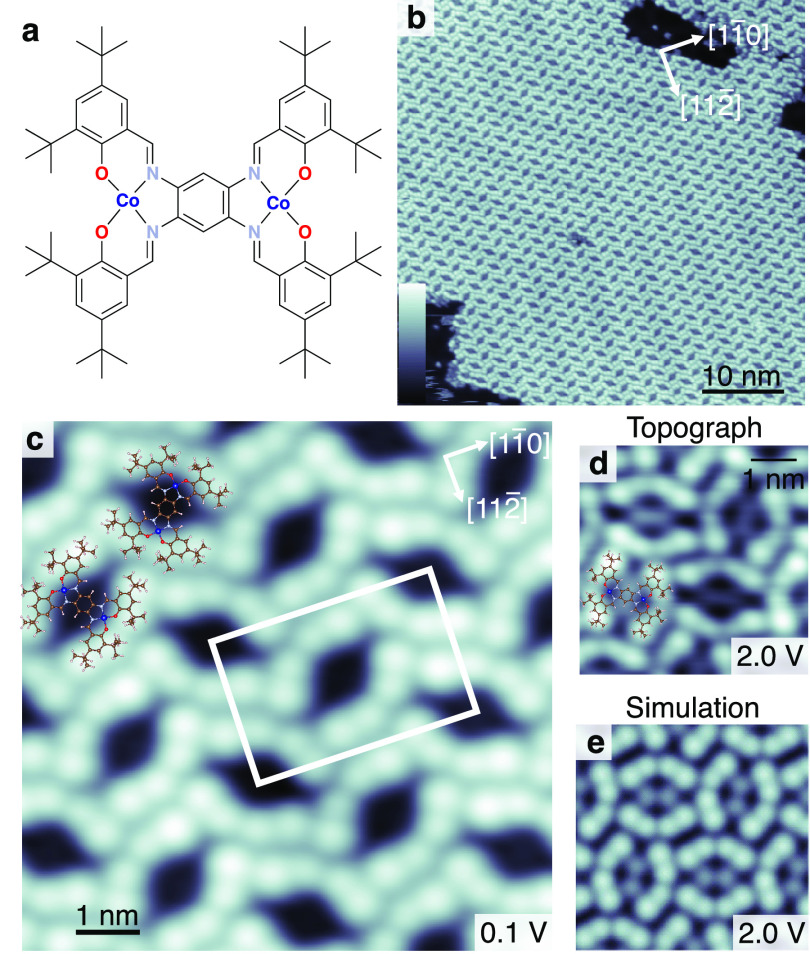
Structure and self-assembly of di-Co on Au(111). (a) Scheme of the investigated di-Co complex. (b) Constant current topograph (0.1 V, 50 pA) of di-Co on Au(111) exhibiting a large island with a regular arrangement of the molecules along with uncovered Au areas (dark). The lower left inset shows the color scale used throughout the manuscript. (c) Detailed image of the molecular arrangement (0.1 V, 50 pA). The indicated unit cell contains two molecules. The primitive vectors (lengths 3.55 and 2.44 nm) enclose an angle of 90°. The substrate directions  (densely packed, oriented along the herringbone reconstruction), and  are indicated with arrows. Two di-Co are overlaid to better visualize the organization of the molecules (dark blue: Co, light blue: N, red: O, brown: C, and pink: H). (d) STM topograph and (e) simulation based on DFT calculations at a sample voltage of 2.0 V. At this voltage, an intramolecular pattern is visible at the center of the molecule.

## Results and Discussion

### Self-Assembly on Au(111)

The complex di-Co is comprised of two Co(salophen) subunits connected by a shared phenyl ring (Figure 1a). Eight *tert*-butyl moieties are located at the periphery of the complex to obtain a degree of decoupling from the substrate.^21,22^

On Au(111), the complex self-assembles into a well-ordered pattern (Figure 1b) with a rectangular unit cell that contains two molecules (Figure 1c). The most prominent image contrast is caused by the bulky *tert*-butyl subunits (see overlaid molecules in Figure 1c) in agreement with DFT calculations. At a sample voltage of 2.0 V, a pattern is visible at the center of the complexes, which is reproduced in the simulated image (Figure 1d,e). Using the *C*_2_ axis of the molecule that is perpendicular to the line connecting the Co(II) ions to define the orientation of the molecule, we observe that the molecules enclose angles of ≈ ±35° with respect to one of the dense directions of the Au substrate ( and ).

### Magnetic Properties

Solid-state susceptibility measurements of di-Co powder using a superconducting quantum interference device (SQUID) have been reported by Shimakoshi et al.^23^ The data indicate that the Co(II) ions each carry a spin 1/2 and couple antiferromagnetically. An exchange energy, defined as the energy difference between the singlet and triplet states, of 2.5 meV was inferred from the data.

**Figure 2:**
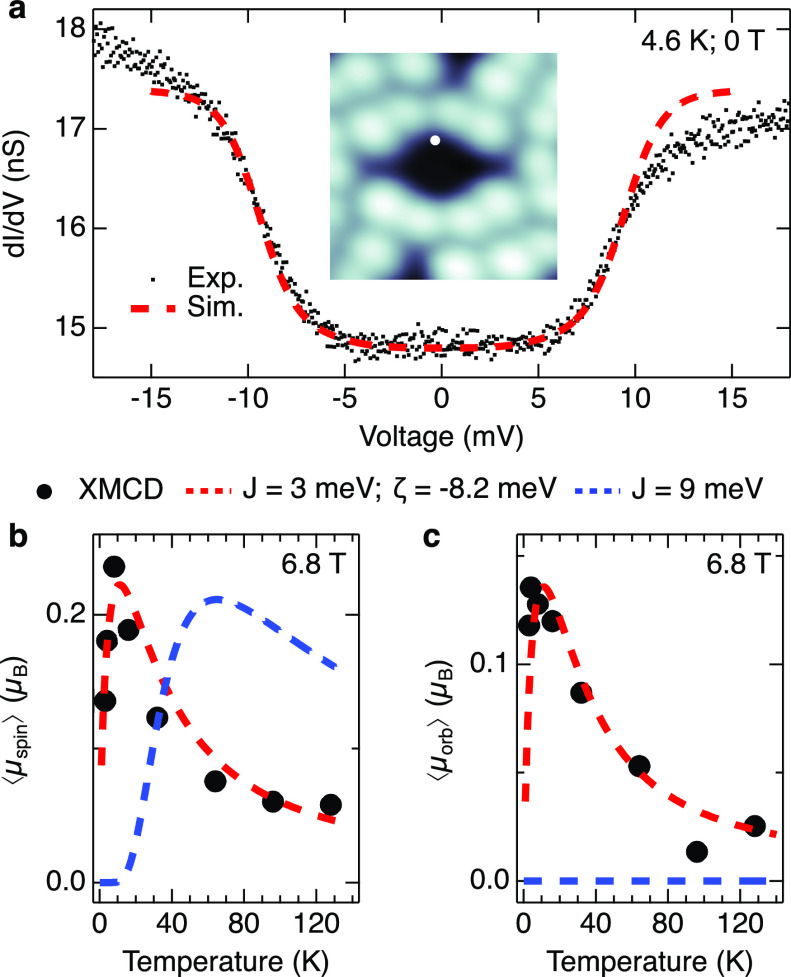
Magnetic excitations of di-Co. (a) d*I*/d*V* spectrum (dots) acquired atop a Co(II) ion exhibiting pronounced steps at *V* ≈ 9 mV, ascribed to magnetic excitations. The experimental data are essentially reproduced with the Hamiltonian of eq 2 including thermal broadening (dashed-dotted red line *J* = 3 meV, ζ = −8.2 meV). The white dot in the inset indicates the position at which the spectrum was acquired. The current feedback loop was disabled at *V* = 30 meV and *I* = 500 pA. Evolution of (b) ⟨μ_spin_⟩ and (c) ⟨μ_orb_⟩ with temperature (black dots) as inferred from XMCD spectra on di-Co powder. The dashed red and blue lines are angle-averaged simulations with and without spin–orbit coupling (Supporting Information Section 3). The simulations without spin–orbit coupling (blue) were done with *J* = 9 meV, which matches the energies of the steps in (a). For better visualization, the red lines have been multiplied by a factor of (b) 0.6 and (c) 0.45, while the blue line has been multiplied by 6. The retrieved ⟨μ_spin_⟩ are known to be affected by a magnetic dipole term (ignored here). The model considers states with μ_orb_ = ± 1 μ_B_ for each Co ion, which is here effectively scaled down to orbital moments of 0.45 μ_B_.

Differential conductance spectra of di-Co on Au(111) exhibit marked steps at |*V*| ≈ 9 mV (with variations of ±2 meV from molecule to molecule using similar tunneling conditions), symmetric about the Fermi level at *V* = 0 (Figure 2a). These steps are only visible in spectra taken in the vicinity of the Co ions (Supporting Information Section 6). The magnetic origin of the steps is confirmed by their conversion into a Kondo resonance upon particular manipulations of the complex as described below. Singlet–triplet transitions of molecules on surfaces have been previously reported, involving the spins of organic compounds,^24−27^ the spin of a metal center coupled to that of a ligand,^28^ the spins of separate molecules,^29^ and the spins of different shells on the same atom.^30^ Magnetic excitations of single molecular magnets on insulating surfaces, where several metal atoms are connected through oxygen atoms, have been observed as well.^19,20^ Therefore, the steps in the d*I*/d*V* spectrum may, at first glance, be interpreted as being due to singlet-to-triplet excitations with an exchange energy of ≈9  meV. However, this energy is much larger than values from solid-state measurements (2.5 meV). This discrepancy indicates that further ingredients may have to be considered.

We performed additional X-ray magnetic circular dichroism (XMCD) measurements (see the “Methods” section) on powder samples, which allow us to separate the spin and orbital contributions to the magnetic moment. XMCD combined with sum rules^31−33^ essentially gives the average projection of spin ⟨μ_spin_⟩ and orbital ⟨μ_orb_⟩ moments per Co(II) ion onto the axis of photon-incidence, along which the magnetic field is applied. These data, acquired under a magnetic field of 6.8 T, are shown in Figure 2b,c for temperatures between 2 and 140 K. The evolution of the spin moment with temperature is not compatible with that of two coupled spins with an exchange energy *J* = 9 meV (dashed blue data). Instead, as shown in the Supporting Information Section 1, the experimental data can be reproduced by considering an exchange energy of 1.3 meV. This value is only half that inferred from the SQUID measurements (2.5 meV) performed under a substantially smaller field of 50 mT.^23^ The apparent discrepancy of the exchange energies again suggests that the underlying assumption of coupled spins is insufficient, despite the fact that it leads to fairly good fits of the experimental data.

In addition, the XMCD data reveal a sizable orbital moment of up to ≈0.15  μ_B_ per Co ion with a strong temperature dependence (Figure 2c). This value represents a lower bound, because XMCD only gives the average projection of the orbital moments along the magnetic-field direction. Importantly, the orbital moment is comparable to the spin moment and can consequently not be neglected. For comparison, an orbital moment of 0.3 μ_B_ is inferred from our DFT calculations. This is again a lower bound as DFT typically underestimates orbital moments.^34^ For di-Co adsorbed on Au(111), an orbital moment between 0.1 and 0.3 μ_B_, depending on the direction of the applied field, is inferred from XMCD (Figure S2), and DFT calculations indicate an orbital moment of ≈0.3  μ_B_. Similar magnitudes of spin and orbital moments are found for di-Co powder and di-Co/Au(111) indicating that the magnetic properties of di-Co are not significantly affected by the adsorption. This is corroborated by essentially identical X-ray absorption spectra for di-Co powder and di-Co/Au(111) (Figure S2).

**Figure 3:**
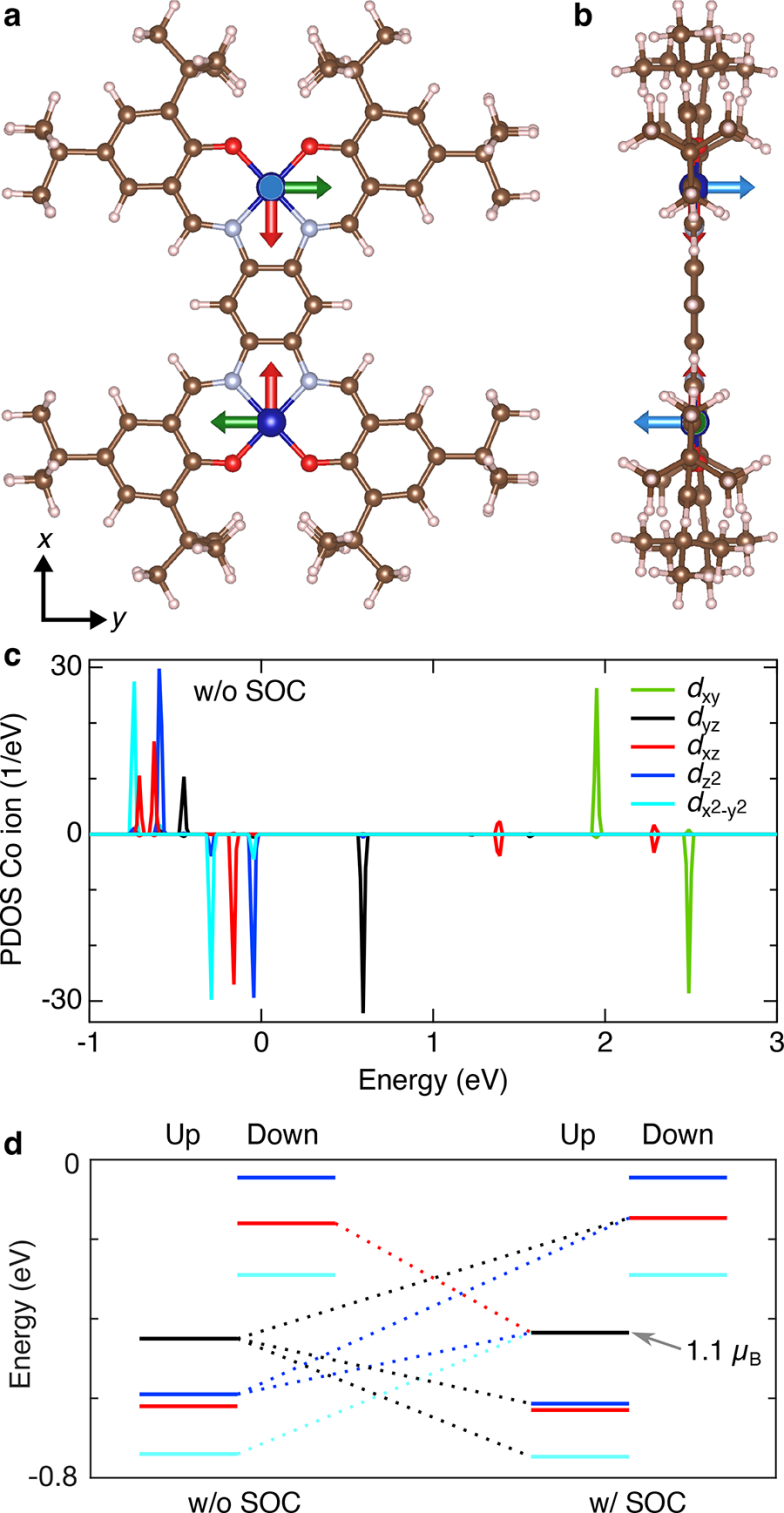
Magnetic anisotropy and origin of the orbital moment. (a) Top and (b) side views of a di-Co complex. According to our DFT calculations in the gas-phase with spin–orbit coupling, the spin configuration shown with red arrows (along the *x* axis) is 3.58 and 3.77 meV lower in energy than the green (*y* axis) and blue (*z* axis) configurations, respectively. Comparable values of 4.17 and 3.08 meV are found for the molecule adsorbed on Au(111). (c) Density of states projected on the d orbitals of the lower Co ion in (a) inferred from our DFT calculation without spin–orbit coupling (*U*_eff_ = 0). The PDOS of the other Co ion is similar to an opposite spin polarization (not shown). (d) Energy diagram of the d orbitals obtained upon hybridization and shift of |*l*, *m*⟩ states (left). The parameters have been adjusted to approximately reproduce the PDOS. SOC hybridizes orbitals (dotted lines) and induces small energy shifts. These calculations predict an orbital moment of 1.1 μ_B_ for the singly occupied d_*yz*_ orbital. The colors of the solid lines indicate the dominant character of the orbital following the legend of (c), while dotted lines highlight admixing of additional orbitals. For clarity, only the largest admixings are represented.

As a consequence of the nonzero orbital moment, the spins of the Co ions, coupled to the orbital moments through SOC, are expected to exhibit anisotropy. This is confirmed experimentally by XMCD measurements of a di-Co monolayer on Au(111). A field of 5 T at 1.5 K is insufficient to turn a detectable fraction of the spins out of the surface plane (⟨μ_spin_⟩ ≈ 0, Supporting Information Section 2). In contrast, measurements at an incidence angle of 60° between the X-rays and the surface normal give ⟨μ_spin_⟩ ≈ 0.2 μ_B_. These measurements show that the spins are preferentially oriented in the molecular plane, which is consistent with our DFT calculations including spin–orbit coupling. The configuration with spins aligned along the axis connecting the two Co ions (red arrows in Figure 3a,b) is ≈4  meV lower in energy than the configurations with spins aligned along perpendicular directions (green and blue arrows in Figure 3a,b).

Having found sizable orbital moments for di-Co in powder samples and adsorbed on Au(111), we next discuss their microscopic origin. The orbital moment of metal–organic compounds is usually quenched by the ligand field. An electron in, e.g., a pure  orbital (represented as |*l*, *m*⟩ with *l* and *m* being the azimuthal and magnetic quantum numbers) or in a pure  orbital is expected to have an orbital moment of zero (⟨d_*yz*_|*L*_*z*_|d_*yz*_⟩ = ⟨d_*xz*_|*L*_*z*_|d_*xz*_⟩ = 0). However, if |d_*yz*_⟩ and |d_*xz*_⟩ states are mixed, e.g., because they are degenerate or through SOC,^35^ then the eigenfunctions are no longer pure Cartesian orbitals. The occupation of  would for instance provide an orbital moment of 1 μ_B_ along the quantization axis *z*. A concomitant occupation of the state  would add an additional moment of −1 μ_B_, thereby quenching the total orbital moment. Similar conclusions can be drawn for the mixing of the d_*xy*_ and  orbitals, which are linear combinations of the |2, +2⟩ and |2, −2⟩ states.

The situation is more complex when the spin quantization axis *z*′ does not coincide with the *z* axis of the molecule. In such cases, the spin–orbit Hamiltonian can be rewritten as^8,36^
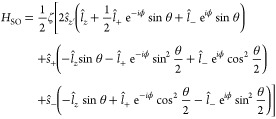
1where , , and  (, , and ) are the *z* (*z*′) component and the ladder operators of the orbital (spin) momentum, ζ is the spin–orbit constant, and θ and ϕ are the polar and azimuthal angles giving the orientation of *z*′ in the (*x*, *y*, *z*) coordinate system used to describe the orbital. In the present case, the *z* axis of di-Co is perpendicular to the molecular plane, while the spin axis appears to be along *x* (Figure 3a) such that θ = π/2 and ϕ = 0. Under these conditions, SOC mixes orbitals whose *l*_*z*_ differ by one (Δ*l*_*z*_ = 1). For example, SOC hybridizes the d_*yz*_ and  orbitals leading to an orbital moment along *x*. The degree of hybridization and hence the magnitude of the moment scales with ζ and inversely with the energy splitting between the d_*yz*_ and  orbitals.

In the following, we consider the impact of *H*_SO_ on di-Co. DFT calculations (gas-phase molecule, *U*_eff_ = 0, and without SOC) find each Co atom in a 4s^2^3d^7^ configuration with a projected density of states PDOS shown in Figure 3c. We then construct a Hamiltonian *H*_Hyb_ in a |*l*, *m*, *s*_*z*_⟩ base, which hybridizes and shifts |*l*, *m*, *s*_*z*_⟩ states with parameters adjusted to approximately reproduce the PDOS (left diagram in Figure 3d). The eigenstates of *H*_Hyb_ + *H*_SO_ are shown in the right part of Figure 3d. SOC induces small energy shifts and hybridization (indicated with dotted colored lines) of the states. For example, the d_*yz*_^↑^ orbital, upon SOC becomes approximately |d_*yz*_^↑^⟩_SOC_ = 0.94|d_*yz*_^↑^⟩ + 0.28*i*|d_*z*^2^_^↑^⟩ – 0.16*i*|d_*xz*_^↓^⟩ + 0.11*i*|d_*x*^2^–*y*^2^_^↑^⟩ with a substantial orbital moment μ_*x*_ = 1.1 μ_B_ (collinear with the spin). We used ζ = 65 meV as reported for Co(II) ions.^35,37^ The compositions and orbital moments of the other orbitals are given in the Supporting Information Section 4. The occupation of other orbitals (7 in total) decreases the total orbital moment to 0.38 μ_B_. This value is in line with the one given by DFT directly (≈0.3  μ_B_). Our calculations show that a large energy splitting due to a ligand field does not necessarily quench the orbital moment.

Having confirmed a significant orbital moment on each Co atoms and explained its origin, we construct a single-electron Hamiltonian to describe the coupling between the two Co atoms in the di-Co molecule that takes into account the effect of the orbital moment and reproduces the experimental data:
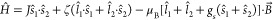
2where the indices 1 and 2 refer to the Co ion sites, *J* is the exchange-coupling constant, *g*_*s*_ the *g*-factor of the spin, and *B⃗* is the external magnetic field. The second term of the Hamiltonian describes spin–orbit coupling of collinear orbital and spin moments (both appear to be along the *x* axis of the molecule). The third term describes the Zeeman energy due to the interaction with the external magnetic field. A number of simplifications are made to reduce the complexity of the model: (i) We use a single-electron Hamiltonian instead of handling the 7 d-electrons per Co ion, such that Coulomb repulsion and correlations are not taken into account. (ii) We solely consider linear combinations of states with *m* = ±1 for each ion site. This is technically achieved by taking *L*_1_ = *L*_2_ = 1 and by shifting the *m* = 0 states 100 meV up in energy. (iii) As simplification (ii) overestimates the orbital moments, the spin–orbit coupling is rescaled by adjusting ζ. The Hamiltonian (eq 2) is diagonalized, and the occupation of the eigenstates is described with a Boltzmann distribution. The spin and orbital moments are then projected along *B⃗*, and these values averaged over the polar and azimuthal angles of *B⃗* for a fixed orientation of the molecule (Supporting Information Section 3). Using *J* = 3 meV and ζ = −8.2 meV, the thermal evolutions of the spin and orbital moments and the differential conductance spectrum are simultaneously reproduced (dashed red curves in Figures 2). Taking into account the variation of the excitation energy observed between molecules, ζ has to be adjusted from ≈−10 to −6 meV to reproduce the d*I*/d*V* spectra. This does not significantly affect the temperature evolutions of ⟨μ_spin_⟩ and ⟨μ_orb_⟩ (Supporting Information Section 8).

With the above parameters and *B* = 0, the degenerate ground state of (in the basis |*m*_1_, *s*_1,*z*_, *m*_2_, *s*_2,*z*_⟩). For d*I*/d*V* spectra, excited states that differ from the ground state by the angular momentum of a tunneling electron are relevant, hence |Δ*m*_*J*_| = 0, 1. These states essentially are linear combinations of |1, *↓*, −1, *↓*⟩, |−1, *↓*, 1, *↓*⟩, |1, *↑*, −1, *↑*⟩, and |−1, *↑*, 1, *↑*⟩. Steps in the d*I*/d*V* spectrum may be understood as a spin flip at one of the Co sites and correspond to a transition from an antiferromagnetic to a ferromagnetic configuration of the Co spins. However, the angular momentum provided by the tunneling electron (maximum of 1) is insufficient to change the orbital moment of the Co sites. Consequently, spin and orbital momentum are antiparallel at the excited spin-flipped Co site. The energy of the excited state has therefore contributions from the unfavorable spin alignment (exchange coupling) and the unfavorable alignment of the spin and orbital momentum at a Co site (due to SOC), which leads to the large excitation energy observed in d*I*/d*V* spectra. In contrast to excitation by electrons, thermal excitation is not limited to |Δ*m*_*J*_| ≤ 1. This results in the evolutions with temperature shown in Figure 2.

### Tuning Magnetic Anisotropy

The magnetic anisotropy of Co(II) ions on surfaces was shown to decrease as the exchange coupling to the substrate is increased.^38−40^ For di-Co on Au(111), we observed variations of the excitation energy (from ≈7 to 11 meV; spectra are acquired with tunneling conditions weakly perturbing the molecule) measured on different complexes. Although a clear pattern has not yet been identified, the electronic coupling presumably depends on the location of the complex relative to the reconstruction of the Au(111) surface.

**Figure 4:**
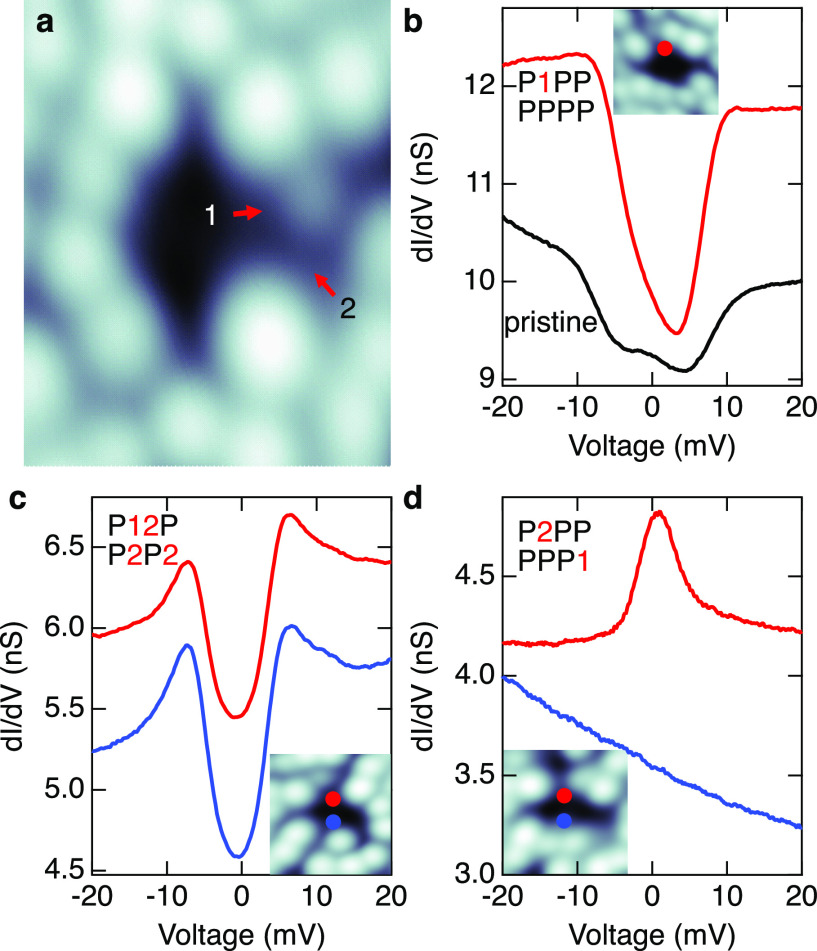
Tuning the magnetic anisotropy through manipulation of *tert*-butyl moieties. (a) Topograph (height range 2.8 nm) of a di-Co complex showing states 1 and 2 (arrows) of the *tert*-butyl moiety that are usually obtained upon injection of current (2.7 V, ≈3  nA). (b) d*I*/d*V* spectra acquired atop a Co center before (black) and after (red) manipulation of the second top-left *tert*-butyl moiety into state 1. The 2 × 4 array of characters describes the states of the eight *tert*-butyl groups (P for pristine). (c) d*I*/d*V* spectra of a complex subject to multiple manipulations. Both Co centers (shown as red and blue spots in the insets) exhibit similar features. (d) d*I*/d*V* spectra of another manipulated complex. The insets of panels (b–d) are topographs of the measured complexes after manipulation, and the dots indicate the position at which the spectra were acquired. Tip height set points: (a) 50 mV, 500 pA, (b) 50 mV, 300 pA, and (c) 30 mV, 100 mV.

To confirm the impact of electronic coupling on the magnetic anisotropy, we manipulated (through current injection) the *tert*-butyl moieties that decouple the Co centers from the surface. Three states of the *tert*-butyl groups (P, 1, and 2) with different apparent heights were obtained (Figure 4a). Changing a single moiety into state 1 shifts the steps toward lower energy by ≈30% (Figure 4b, red vs black curve). Modification of more *tert*-butyl moieties further decreases the energies of the steps and leads to broad peaks at the edges of the excitation gap (Figure 4c). Some molecules exhibit a peak with Frota line shape (Figure 4d) typical of a Kondo resonance^41,42^ instead of gap-like spin excitations. When a Kondo resonance is observed atop one Co center, the other one does not show excitation steps. Its spectrum is either featureless (blue curve in Figure 4d) or exhibits a Kondo resonance as well.

**Figure 5:**
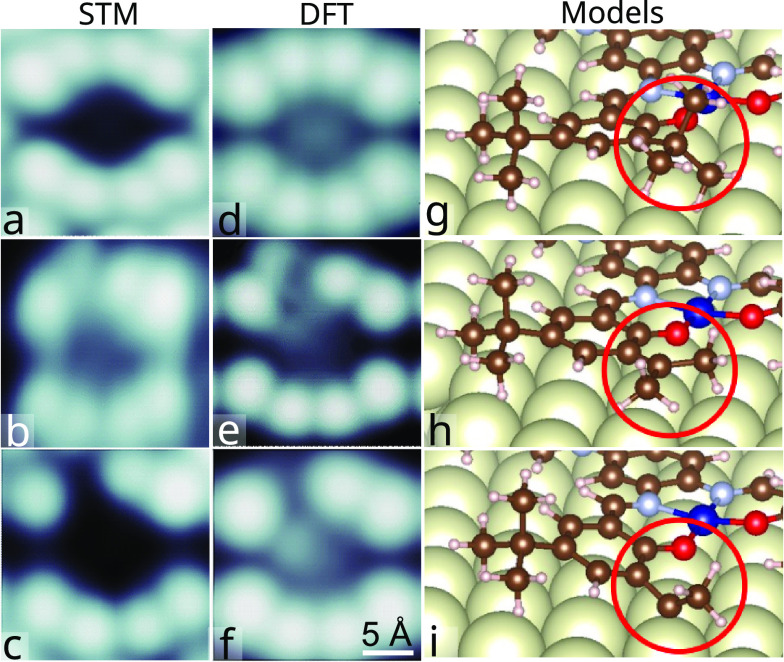
Manipulation of *tert*-butyl: STM and models. (a–c) Experimental STM images (*V* = 0.1 V, *I* = 0.1 nA) of a di-Co complex. The initial state shown in (a) was modified by injecting current (*V* = 2.7 V, *I* = 3 nA, duration 10 s) into one of the *tert*-butyl groups which led to a changed image contrast in (b). The procedure was subsequently repeated on the same molecular subunit leading to the image shown in (c). The apparent height of the molecule in this area is further reduced. (d–f) Images calculated for the models (g–i) approximately reproduce the experimental images. The removal of one (h) or two (i) methyl groups induces an increasing amount of distortion to the complex. In particular, the C atom from which methyl has been striped off is closer to the substrate to compensate for the lacking ligand.

The manipulation of the *tert*-butyl groups may be interpreted in terms of sequential removal of methyl groups. The corresponding simulated STM images match the experimental ones quite well (Figure 5). Our DFT calculations reveal energy shifts of the d orbitals upon manipulation of the *tert*-butyl moieties. The energy of the d orbitals is important for hybridization and the magnitude of the orbital moment, as illustrated in Figure 3. This is confirmed in our calculations, where the orbital moment of the Co site close to the manipulated ligands decreases with the number of removed methyl groups (0.311, 0.278, and 0.269 μ_B_ for 0, 1, and 2 abstracted methyl groups, respectively), while that of the other Co site remains fairly constant (0.295, 0.302, and 0.298 μ_B_). In addition, the interaction with the substrate appears to affect the exchange coupling between the two sites as well. We speculate that the removal of further methyl groups further decreases the orbital moment and the exchange coupling. The Co ions may then be described as pure spin-1/2 systems interacting with the conduction electrons of the substrate, leading to a Kondo resonance. The change of orbital moment and exchange coupling affects the magnetic anisotropy. Orienting the spins along the *y* axis (green arrows in Figure 3a) costs respectively 4.17, 3.29, and 2.72  meV more energy than along the *x* axis (Supporting Information Section 5). That is, the magnetic anisotropy of the molecule is tuned by manipulating the *tert*-butyl moieties.

**Figure 6:**
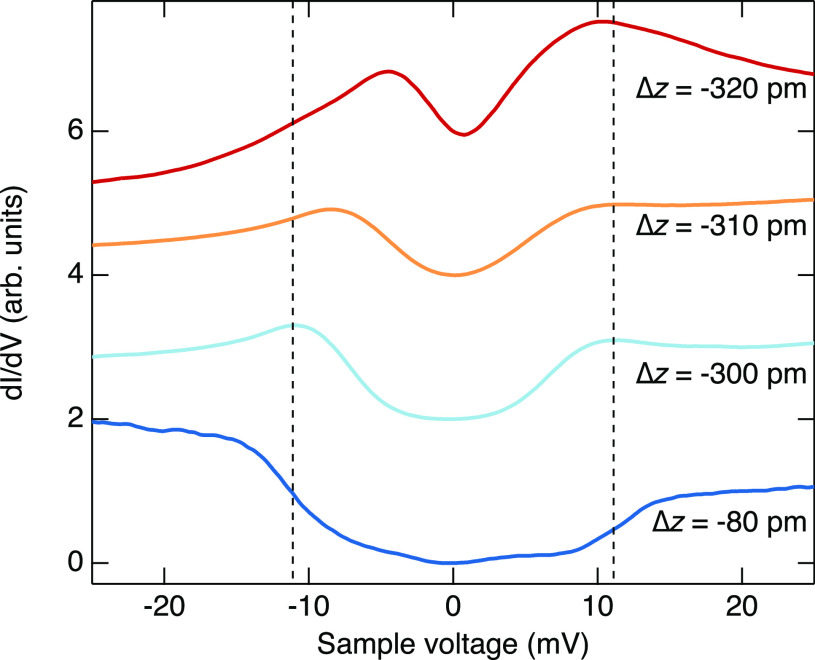
Evolution of spin-excitation energy with tip-to-Co distance. d*I*/d*V* spectra acquired at different relative heights over a Co(II) ion of di-Co. A tip height Δ*z* = 0 is defined at tunneling parameters *V* = 40 mV and *I* = 100 pA. Δ*z* becomes more and more negative as the tip approaches the molecule. Δ*z* = −80 and −320 pm respectively correspond to initial currents of *I* = 0.5 and 550 nA (*V* = 40 meV). The vertical lines are guides to the eye indicating the positions of the steps of the lowest spectrum (Δ*z* = −80 pm).

The manipulation of the *tert*-butyl subunits allows a relatively coarse modification of the magnetic properties with limited control over the final state. Fine-tuning of the magnetic anisotropy is possible by bringing the STM tip close to a Co center. Figure 6 shows data from a pristine molecule recorded at various vertical tip displacements Δ*z*. The magnetic excitation energy of ≈11 meV for Δ*z* ≥ – 80 pm decreases as Δ*z* becomes more negative, while the d*I*/d*V* increases at the steps developing overshoots. This effect may be attributed to an increased interaction of the Co center with the conduction electrons of the tip and the substrate. Further data and fits are shown in Supporting Information Section 7.

## Conclusions

In conclusion, we found that the two *S* = 1/2 spins separated by a benzenetetraimine moiety of a di-Co complex on Au(111) are antiferromagnetically coupled. Differential conductance spectra, XMCD measurements, and DFT calculations reveal large orbital moments comparable to the spin moments despite the ligand field acting on the Co ions. The orbital moments, often neglected for spin 1/2 ions, lead in turn to a sizable magnetic anisotropy. The orbital moment and the magnetic anisotropy, may be tuned by manipulating the peripheral groups of the complex or by bringing the STM tip close the molecule. Our study shows that unquenched orbital moments are relevant in transition metal complexes and lead to sizable magnetic anisotropies even for spin-1/2 objects.

## Methods

### Synthesis

The di-Co powder was synthesized following the description of ref (23).

### STM

The Au(111) surface was prepared by cycles of Ar ion bombardment (1.5 keV) and annealing to 450 °C. di-Co was sublimated from a heated crucible (≈260 °C) onto the substrate at ≈25 °C. STM tips were electrochemically etched from W wire and annealed *in vacuo*. Experiments were carried out in ultrahigh vacuum with a STM operated at ≈4.6 K. The differential conductance d*I*/d*V* was measured using a lock-in amplifier with a modulation voltage of 0.5 mV_*rms*_ at 667.8 Hz. The shown d*I*/d*V* spectra were low-pass filtered.

### XMCD

Measurements on di-Co powder pressed onto a Ta foil were performed at the EPFL/PSI X-Treme beamline^43^ at the Swiss light source (Proposal 20190693). X-ray absorption spectra at the Co *L*_3,2_ edges were acquired under a magnetic field of 6.8 T at different temperatures. The difference of such spectra, recorded with photons of left and right helicities, gives the XMCD spectra. Sum rules have been applied to the spectra to extract the average spin and orbital moments aligned along the magnetic field.^33^ A 3d^7^ occupation of the Co ions has been used. The X-ray absorption measurements on di-Co on Au(111) (sample prepared as for STM measurements) were performed at the DEIMOS beamline^44^ of the synchrotron SOLEIL (proposal 20190691).

### DFT

Density functional theory (DFT) calculations were performed using the VASP code.^45^ Core electrons were treated using the projected augmented-wave (PAW) method^46^ and wave functions were expanded using a plane wave basis set with an energy cutoff of 400 eV. The PBE was used as exchange and correlation functional.^47^ The description of the Co d-electrons was improved by using the GGA+U method as formulated by Dudarev^48^ with *U*_eff_ = 3 eV if not otherwise stated. Missing long-range dispersion interactions in this functional were treated using the Tkatchenko–Scheffler method..^49^ Magnetic anisotropy energies were calculated by performing total energy differences between different configurations after including spin–orbit coupling as implemented in VASP.^50^

The Au(111) surface was simulated using the slab method with four atomic layers separated by a vacuum region of 21 Å. The coordinates of all atoms except the two bottom layers were relaxed until forces were smaller than 0.02 eV/Å.

STM simulations were done by applying the Tersoff–Hamann approximation^51^ using the method of Bocquet et al.^52^ as implemented in the code STMpw.^53^

## Data Availability

Raw data may be obtained from the corresponding authors upon reasonable request.

## Supporting Information Available

Spin–spin coupling, XAS and XMCD on di-Co on Au(111), simulation of the projected magnetic moment, mixing of d orbitals, the manipulation of *tert*-butyl groups, spatial extent of the magnetic excitations, fits of d*I*/d*V* spectra, and influence of the spin–orbit coupling constant (PDF)
