# Supplementary material for: Large Orbital Moment of Two Coupled Spin-Half Co Ions in a Complex on Gold
Source: ACS Nano. 2023 May 24;17(11):10608–16. doi: 10.1021/acsnano.3c01595 (PMC10278185; doi:10.1021/acsnano.3c01595)
Supplement: Supplementary file 1 — nn3c01595_si_001.pdf [file nn3c01595_si_001.pdf]

Supporting Information: Large Orbital Moment of Two Coupled Spin-Half Co Ions in a Complex on Gold

Chao Li,[†] Roberto Robles,[*,‡] Nicolas Lorente,[‡,¶] Sanjoy Kr Mahatha,[§] Sebastian Rohlf,[†] Kai Rossnagel,[†,§] Alessandro Barla,[∥] Boris V. Sorokin,[⊥] Stefano Rusponi,[⊥] Philippe Ohresser,[#] Sara Realista,[@] Paulo N. Martinho,[@] Torben Jasper-Toennies,[†] Alexander Weismann,[†] Richard Berndt,[*,†] and Manuel Gruber[*,△]

†Institut für Experimentelle und Angewandte Physik, Christian-Albrechts-Universität zu Kiel, 24098 Kiel, Germany

‡Centro de Física de Materiales CFM/MPC (CSIC-UPV/EHU), 20018 Donostia-San Sebastián, Spain

¶Donostia International Physics Center (DIPC), 20018 Donostia-San Sebastian, Spain

§Ruprecht Haensel Laboratory, Deutsches Elektronen-Synchrotron DESY, 22607 Hamburg, Germany

∥Istituto di Struttura della Materia (ISM), Consiglio Nazionale delle Ricerche (CNR), 34149 Trieste, Italy

⊥Institute of Physics, Ecole Polytechnique Fédérale de Lausanne (EPFL), Station 3, 1015 Lausanne, Switzerland

#Synchrotron SOLEIL, L'Orme des Merisiers, 91190 Saint Aubin, France

@Centro de Química Estrutural, Institute of Molecular Sciences, Departamento de Química e Bioquímica, Faculdade de Ciências, Universidade de Lisboa, Campo Grande, 1749-016 Lisboa, Portugal

△Faculty of Physics and CENIDE, University of Duisburg-Essen, 47057 Duisburg, Germany

E-mail: roberto.robles@csic.es; berndt@physik.uni-kiel.de; manuel.gruber@uni-due.de

1 Spin-spin coupling

Below, our XMCD data are analyzed neglecting spin-orbit coupling. We use a spin Hamiltonian describing the exchange coupling between the spins of the two ions and a Zeeman energy

$$H_{\text{spin}} = J\hat{s}_1 \cdot \hat{s}_2 - \mu_{\text{B}} g_s(\hat{s}_1 + \hat{s}_2) \cdot \vec{B}, \qquad \text{(S1)}$$

where $\hat{s}_1$ and $\hat{s}_2$ are the spin operators ($S = 1/2$) at the ion sites. J is the exchange coupling constant, g_s the spin g-factor and $\vec{B}$ the magnetic field. The occupation of the eigenstates is then determined with Boltzmann statistics. Figure S1 shows the evolution of the average spin moment per Co ion as a function of temperature for different J values. The best agreement with the data is obtained for $J = 1.3\,\text{meV}$ (red curve). As stated in the manuscript, this analysis is incorrect albeit leading to a good fit.

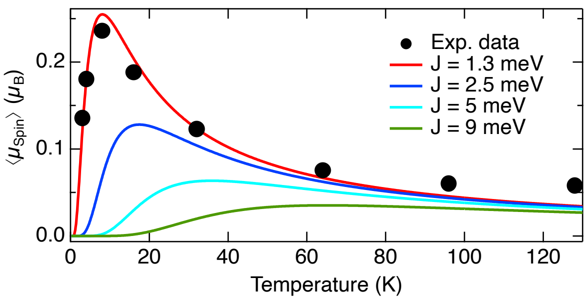

Figure S1: Evolution of the spin moment per Co ion inferred from XMCD as a function of temperature (black dots). The colored curves are from the spin Hamiltonian with different J as indicated in the legend.

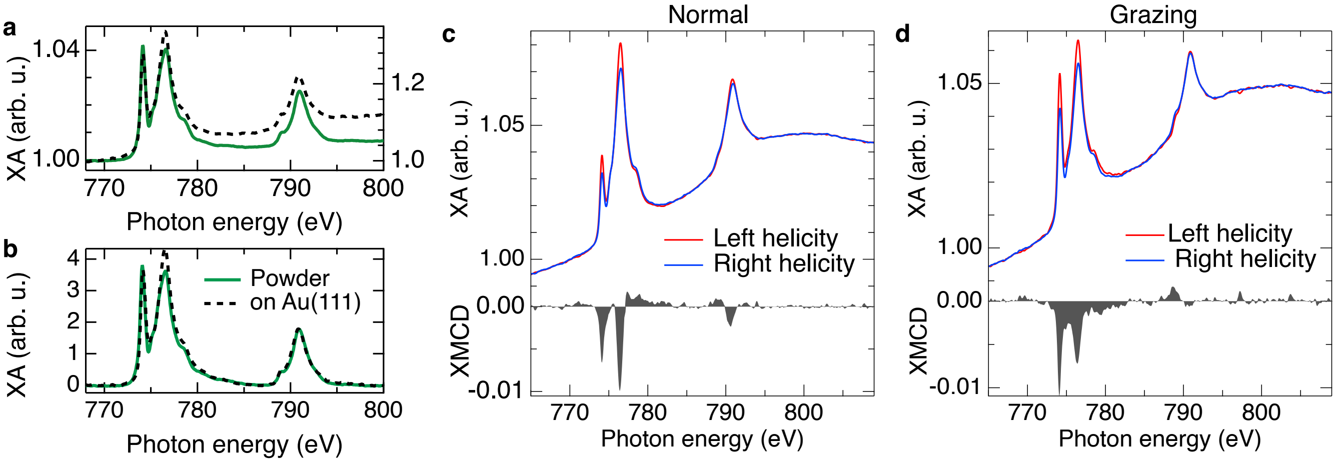

Figure S2: XA spectra of di-Co powder (green line) and of ≈ 1 ML di-Co on Au(111) (dashed black line) at room temperature **a** before and **b** after background subtraction and scaling. The spectra are averages of four sweeps. XA and XMCD spectra of ≈ 1 ML di-Co on Au(111) acquired under **c** normal and **d** grazing incidence in a field of 5 T and at 1.5 K. Sum rules applied to the spectra yield **c** $\langle m_s \rangle \approx 0$, $\langle m_l \rangle \approx 0.1\,\mu_\mathrm{B}$ and **d** $\langle m_s \rangle \approx 0.2\,\mu_\mathrm{B}$, $\langle m_l \rangle \approx 0.3\,\mu_\mathrm{B}$. The XMCD spectra were obtained from eight XA sweeps acquired with different photon helicities and field directions. As the measurements were carried out on different beamlines, the green spectra in **a**–**b** have been shifted by ≈ -2 eV to match the dashed curve.

2 XAS and XMCD on di-Co on Au(111)

Figure S2b shows that the x-ray absorption (XA) spectrum of ≈ 1 ML di-Co on Au(111) (dashed curve) essentially exhibits the same features as that of di-Co powder. This suggests that the oxidation state and the spin states of the Co(II) ion are not modified by the adsorption on Au(111). XMCD data from the 1 ML sample strongly depend on the photon incidence angle. Under normal incidence (magnetic field perpendicular to the surface), sum rules[S1–S3] lead to $\langle m_s \rangle \approx 0$, whereas under grazing incidence, we find $\langle m_s \rangle \approx 0.2\,\mu_\mathrm{B}$. These results indicate that a magnetic field of 5 T is insufficient to rotate a fraction of the spins out of the molecular plane at 1.5 K, whereas a significant fraction may be aligned in-plane under similar conditions. The observations are consistent with the magnetic anisotropy inferred from our powder measurements and DFT calculations.

3 Simulation of the projected magnetic moment

The Hamiltonian of Equation 1 of the main manuscript is diagonalized in the $|m_1, S_{1,z}, m_2, S_{2,z}\rangle$ basis leading to N eigenvalues E_i and eigenvectors V_i ($N = 100$ with $L_1 = L_2 = 2$ and $S_1 = S_2 = 1/2$). The expectation value of an operator $\hat{X}$ at a given temperature reads

$$\langle X \rangle = \sum_{i=0}^{N-1} \frac{\exp\left(\frac{E_i - E_0}{k_B T}\right)}{Z} V_i^\dagger \hat{X} V_i, \qquad \text{(S2)}$$

where k_B is the Boltzmann constant and Z the partition function.

XMCD is sensitive to the projection of the spin and orbital momenta on the photon-incidence axis (equivalent to the magnetic field axis in the experiment). For polar and azimuthal angles θ and ϕ between $\vec{B}$ and the z axis of a molecule, the measured spin moment reads

$$m_s(\theta, \phi) = 2 \begin{pmatrix} \sin\theta\cos\phi \\ \sin\theta\sin\phi \\ \cos\theta \end{pmatrix} \cdot \begin{pmatrix} \langle S_{1,x}\rangle + \langle S_{2,x}\rangle \\ \langle S_{1,y}\rangle + \langle S_{2,y}\rangle \\ \langle S_{1,z}\rangle + \langle S_{2,z}\rangle \end{pmatrix}. \qquad \text{(S3)}$$

As molecules in powder are randomly oriented, the moment effectively measured is

$$\begin{aligned} \langle m_s \rangle &= \tfrac{1}{2\pi^2} \int_{\theta=0}^{\pi} \int_{\phi=0}^{2\pi} m_s(\theta,\phi) d\theta d\phi \qquad \text{(S4)} \\ &= \tfrac{2}{\pi} \int_{\theta=0}^{\pi/2} m_s(\theta,\phi) d\theta. \end{aligned}$$

Equivalent considerations give the average orbital moment $\langle m_l \rangle$. Simulations integrated over the polar angle θ have been carried out to reproduce the temperature-dependent measurements shown in Figure 2 of the main manuscript.

4 Mixing of *d* orbitals

As explained in the manuscript, we constructed a Hamiltonian H_Hyb mixing and shifting $|l, m, s_z\rangle$ states, where the parameters have been adjusted to approximately reproduce the PDOS inferred from DFT calculations. The spin-orbit Hamiltonian H_SO was then added to H_Hyb and the corresponding eigenstates calculated. The projection of the eigenstates ($|n\rangle$ with $n = 1, .., 10$) on the d orbitals are shown in Table S1 along with the energy and orbital moments of the states.

Table S1: **Composition of the eigenstates with SOC.** E and $\langle \mu_x \rangle$ are the energy and the orbital magnetic moment along x, respectively. Components with coefficients smaller than 0.01 are neglected for clarity. For example, the fourth state $|4\rangle = 0.11|d^{\uparrow}_{x^2-y^2}\rangle + 0.28|d^{\uparrow}_{z^2}\rangle + 0.94i|d^{\uparrow}_{yz}\rangle - 0.16|d^{\downarrow}_{xz}\rangle + 0.01i|d^{\downarrow}_{xy}\rangle$ has an energy of -0.435 eV with respect to the Fermi level and an orbital momentum $\langle 4|\hat{l}_x|4\rangle = -1.14$.

State	$\|1\rangle$	$\|2\rangle$	$\|3\rangle$	$\|4\rangle$	$\|5\rangle$	$\|6\rangle$	$\|7\rangle$	$\|8\rangle$	$\|9\rangle$	$\|10\rangle$
E (eV)	-0.747	-0.630	-0.614	-0.435	-0.290	-0.147	-0.045	0.596	1.953	2.492
$\langle \mu_x \rangle (\mu_B)$	0.24	0.02	0.95	-1.14	-0.07	-0.07	-0.31	0.38	-0.02	0.02
$d^{\uparrow}_{x^2-y^2}$	-0.99	0	0.05	0.11	0	-0.05	0	0	0	-0.02
$d^{\uparrow}_{xz}$	0	0.99	0	0	-0.09	0	0.09	-0.03	0.01	0
$d^{\uparrow}_{z^2}$	-0.02	0	-0.95	0.28	0	0.14	0	0	0	0
$d^{\uparrow}_{yz}$	$-0.12i$	0	$-0.30i$	$0.94i$	0	$-0.13i$	0	0	0	$0.01i$
$d^{\uparrow}_{xy}$	0	$0.01i$	0	0	$-0.03i$	0	0	$0.02i$	$-1.00i$	0
$d^{\downarrow}_{x^2-y^2}$	0	-0.10	0	0	-0.99	0	0.02	0.03	0.03	0
$d^{\downarrow}_{xz}$	-0.06	0	0.10	-0.16	0	0.98	0	0	0	0.01
$d^{\downarrow}_{z^2}$	0	0.09	0	0	-0.03	0	-0.99	0.09	0	0
$d^{\downarrow}_{yz}$	0	$0.02i$	0	0	$0.03i$	0	$0.09i$	$0.99i$	$0.02i$	0
$d^{\downarrow}_{xy}$	$-0.02i$	0	0	$0.01i$	0	$-0.01i$	0	0	0	$1.00i$

5 Manipulation of *tert*-butyl groups

Table S2 shows the magnetocrystalline anisotropy energy as a function of the number of abstracted methyl groups. The calculations confirm the experimentally observed trend of reduced anisotropy energy with the number of manipulations (see for instance ΔE_y).

Table S2: **Influence of methyl abstraction on the magnetic properties.** $E_{FM} - E_{AF}$ is the energy difference between ferromagnetic and antiferromagnetic alignments of the spins. E_i is the energy cost when the spins are along the i axis. All energies are given in meV.

removed methyls	$E_{FM} - E_{AF}$	E_x	E_y	E_z
0	3.1	0	4.17	3.08
1	0.5	0	3.29	3.45
2	1.9	0	2.72	2.55

6 Spatial extent of the magnetic excitations

Figure S3 shows the evolution of the dI/dV spectra along the Co-Co axis of a molecule (red squares). Magnetic excitations are only observed in the vicinity of the Co ions (spectra in red), while the spectra acquired at other positions are essentially featureless (spectra in black). This set of data highlights the central role of the Co ions for the magnetic properties.

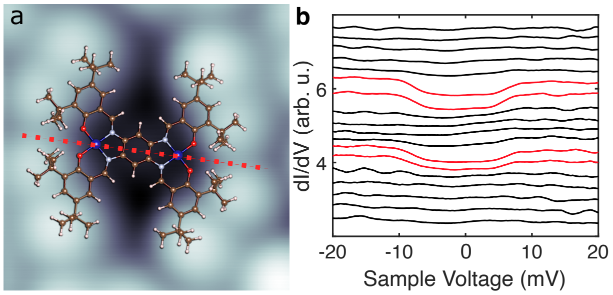

Figure S3: **a** STM image of a di-Co complex with a superimposed molecular model (2 nm wide, $V = 100$ mV, $I = 100$ pA). **b** dI/dV spectra acquired at the positions of the red squares in **a** (bottom to top spectra correspond to left to right squares). The spectra highlighted in red exhibit steps due to magnetic excitation. The feedback was opened at $V = 50$ mV, $I = 500$ pA and the spectra are vertically shifted for clarity.

7 Fits of dI/dV spectra

Differential-conductance spectra were fitted with temperature-broadened step functions and Frota functions at the positions of the steps[S4] in addition to a cubic background:

$$\sigma = \sigma_0 + aV + bV^2 + cV^3 + h_0^- \Theta\left(\frac{E_0 + eV}{k_B T}\right) + h_0^+ \Theta\left(\frac{E_0 - eV}{k_B T}\right) + h_K^-\, g\left(E_0 + eV, \Gamma_K\right) + h_K^+\, g\left(E_0 - eV, \Gamma_K\right), \tag{S5}$$

where Θ is a temperature-broadened step function:

$$\Theta(x) = \frac{1 + (x-1)\exp(x)}{(\exp(x) - 1)^2}, \tag{S6}$$

and g the Frota function:[S5,S6]

$$g(E, \Gamma_K) = \Im \left[i e^{i\phi} \sqrt{\frac{i\Gamma_K}{E + i\Gamma_K}} \right]. \tag{S7}$$

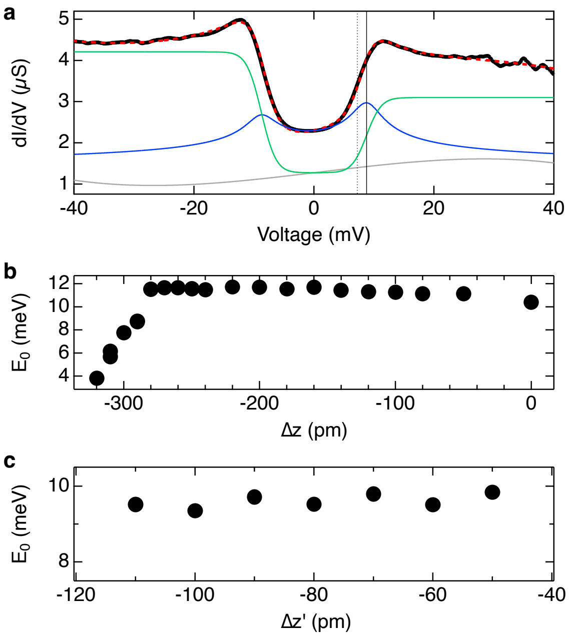

Figure S4: **a** dI/dV spectrum acquired atop a Co(II) ion (black) along with a fit with Equation S5 (red) giving $E_0 = 8.78 \pm 0.06$ meV, $\Gamma_K = 2.60 \pm 0.13$ meV, and $T = 9.44 \pm 0.16$ K. The green, blue and gray curves respectively show the spin-excitation, Frota, and background components of the fit. The vertical solid line indicates the energy E_0, while the dashed vertical line highlights the effective energy of the step. The spectrum is acquired at a relative height $\Delta = -290$ pm. **b** Spin-excitation energy E_0 extracted from dI/dV spectra taken at various relative heights Δz above a Co center. The confidence intervals inferred from the fits are smaller than the size of the points. The height reference $\Delta z = 0$ is obtained when tunneling with $V = 40$ mV and $I = 100$ pA. $\Delta z = -80, -280, -320$ pm respectively correspond to initial currents of $I = 0.5, 130, 550$ nA ($V = 40$ meV). **c** E_0 versus $\Delta z'$ acquired over another molecule with a different tip. The height reference $\Delta z' = 0$ is obtained when tunneling with $V = 50$ mV and $I = 100$ pA.

Fits of dI/dV spectra with Equation S5 reproduce the main structures of the spectra. An example is shown in Figure S4a (dI/dV in black, fit in red). The additional curves in gray, green, and blue, respectively, represent the contributions of the background, the temperature-broadened step functions, and the Frota functions.

When Frota-like overshoots are present, the positions of the dI/dV steps (example shown by dotted vertical line) do not directly correspond to the magnetic-excitation energy E_0 (solid vertical line). As evident from Figure S4a, the Frota contributions effectively reduce the gap width. When the amplitudes of the two Frota functions differ, the gap actually becomes asymmetric about the Fermi level.

Spectra were acquired atop a Co(II) ion for different relative heights. The spectra of Figure 4 of the main manuscript, along with other intermediate measurements, have been fitted using Equation S5. The resulting E_0 are plotted in Figure S4b as a function of the relative height Δz. We observe that values of E_0 drastically decrease from $\Delta z = -280$ to -320 pm as the tip approaches the Co ion.

Although the decrease of E_0 for $\Delta z = -300$ pm is relatively clear, the apparent increase of E_0 from $\Delta z = 0$ to -100 pm (Figure S4b) should be interpreted with caution. Similar data acquired over another molecule with a different tip suggest that E_0 is constant between $\delta z = -110$ and -50 pm (Figure S4c). The difference between the data sets indicates that the uncertainty of E_0 is likely on the order of ± 1 meV and hence larger than the confidence intervals of the adjustable model parameters ($\approx \pm 0.1$ meV).

8 Influence of the spin-orbit coupling constant

The excitation energies in dI/dV spectra for tunneling conditions close to $\Delta z = 0$ vary from ≈ 7 to 11 meV from molecule to molecule. The different spectra can be reproduced by adjusting ζ without significantly affecting the temperature evolutions of $\langle \mu_{\rm spin} \rangle$ and $\langle \mu_{\rm orb} \rangle$ (Figure S5).

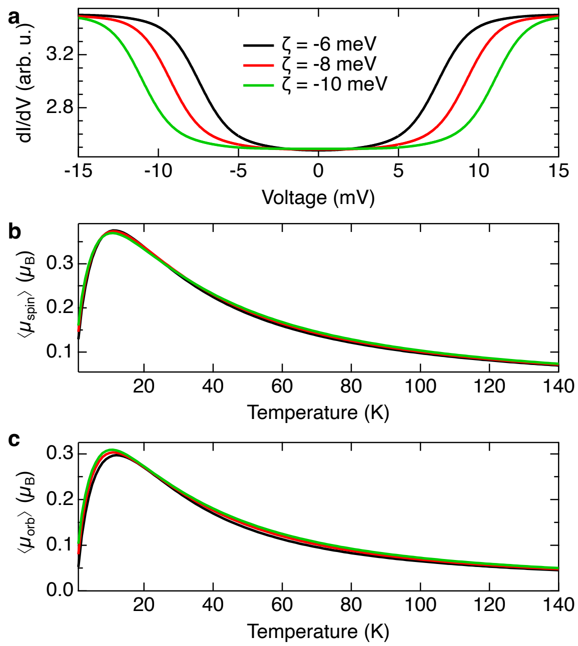

Figure S5: Simulated dI/dV spectra using Equation 2 of the main manuscript with $J = 3$ meV and $\zeta = -6, -8, -10$ meV. Simulated temperature dependencies of **b** $\langle \mu_{\rm spin} \rangle$ and **c** $\langle \mu_{\rm orb} \rangle$ using the same parameters as in **a**.

References

(S1) Thole, B. T.; Carra, P.; Sette, F.; van der Laan, G. X-ray circular dichroism as a probe of orbital magnetization. *Phys. Rev. Lett.* **1992**, *68*, 1943–1946.

(S2) Carra, P.; Thole, B. T.; Altarelli, M.; Wang, X. X-ray circular dichroism and local magnetic fields. *Phys. Rev. Lett.* **1993**, *70*, 694–697.

(S3) Chen, C. T.; Idzerda, Y. U.; Lin, H. J.; Smith, N. V.; Meigs, G.; Chaban, E.; Ho, G. H.; Pellegrin, E.; Sette, F. Experimental confirmation of the X-ray magnetic circular dichroism sum rules for iron and cobalt. *Phys. Rev. Lett.* **1995**, *75*, 152–155.

(S4) von Bergmann, K.; Ternes, M.; Loth, S.; Lutz, C. P.; Heinrich, A. J. Spin Polarization of the Split Kondo State. *Phys. Rev. Lett.* **2015**, *114*, 076601.

(S5) Prüser, H.; Wenderoth, M.; Weismann, A.; Ulbrich, R. G. Mapping Itinerant Electrons around Kondo Impurities. *Phys. Rev. Lett.* **2012**, *108*, 166604.

(S6) Gruber, M.; Weismann, A.; Berndt, R. The Kondo resonance line shape in scanning tunnelling spectroscopy: instrumental aspects. *J. Phys.: Condens. Matter* **2018**, *30*, 424001.
